# CD40-activated B cells induce anti-tumor immunity *in vivo*

**DOI:** 10.18632/oncotarget.7720

**Published:** 2016-02-25

**Authors:** Kerstin Wennhold, Tanja M. Weber, Nela Klein-Gonzalez, Martin Thelen, Maria Garcia-Marquez, Geothy Chakupurakal, Anne Fiedler, Hans A. Schlösser, Rieke Fischer, Sebastian Theurich, Alexander Shimabukuro-Vornhagen, Michael von Bergwelt-Baildon

**Affiliations:** ^1^ Cologne Interventional Immunology (CII), Department I of Internal Medicine, University Hospital of Cologne, Cologne, Germany; ^2^ Department of Hematology, Vall d’Hebron University Hospital, Vall d’Hebron Research Institute (VHIR), Universitat Autònoma de Barcelona, Barcelona, Spain; ^3^ Department of General, Visceral and Cancer Surgery, University Hospital of Cologne, Cologne, Germany; ^4^ Department I of Internal Medicine, University Hospital of Cologne, Cologne, Germany; ^5^ Laboratory for Neuronal Control of Metabolism, Max Planck Institute for Metabolism Research Cologne, Cologne, Germany

**Keywords:** cellular adjuvant, CD40-activated B cells, cancer immunotherapy, antigen presentation, B cell

## Abstract

The introduction of checkpoint inhibitors represents a major advance in cancer immunotherapy. Some studies on checkpoint inhibition demonstrate that combinatorial immunotherapies with secondary drivers of anti-tumor immunity provide beneficial effects for patients that do not show a strong endogenous immune response. CD40-activated B cells (CD40B cells) are potent antigen presenting cells by activating and expanding naïve and memory CD4^+^ and CD8^+^ and homing to the secondary lymphoid organs. In contrast to dendritic cells, the generation of highly pure CD40B cells is simple and time efficient and they can be expanded almost limitlessly from small blood samples of cancer patients. Here, we show that the vaccination with antigen-loaded CD40B cells induces a specific T-cell response *in vivo* comparable to that of dendritic cells. Moreover, we identify vaccination parameters, including injection route, cell dose and vaccination repetitions to optimize immunization and demonstrate that application of CD40B cells is safe in terms of toxicity in the recipient. We furthermore show that preventive immunization of tumor-bearing mice with tumor antigen-pulsed CD40B cells induces a protective anti-tumor immunity against B16.F10 melanomas and E.G7 lymphomas leading to reduced tumor growth. These results and our straightforward method of CD40B-cell generation underline the potential of CD40B cells for cancer immunotherapy.

## INTRODUCTION

A major advance in cancer immunotherapy has been the development of checkpoint inhibitors, which show clinical efficacy in a variety of advanced malignancies [1–4]. Some studies point to the idea that the expression of inhibitory ligands, such as CTLA-4 or PD-L1, by the tumor represents an adaptive response to tumor-specific T cells in the microenvironment [5, 6]. Thus, PD-L1 expression may indicate the presence of an endogenous anti-tumor immunity, while low expression may indicate the absence of such a response [7]. Treatment with a second driver of anti-tumor immune responses was shown to enhance the adaptive induction of the checkpoint molecule PD-L1 on tumor cells [7]. Although contradictory at first sight, this upregulation of checkpoint molecules was critical for the efficacy of the subsequent treatment with checkpoint inhibitors and finally resulted in regression of established tumors. Possible other secondary inducers of anti-tumor immunity might include cellular vaccines or chimeric antigen receptor T cells.

As cellular vaccine, dendritic cells (DCs) have been the major object of interest in pre-clinical studies and clinical trials over the past decade [8–11]. However, after more than 200 clinical studies with DC-based vaccines, the response-rate to vaccination has been disappointingly low. The recent meta-analysis by Draube et al. included 29 clinical trials in patients with prostate and renal cell cancer and demonstrated a statistically significant association between the DC-induced cellular immune response and the clinical outcome [9]. However, this meta-analysis also identified several challenges and areas of uncertainty: First, the ideal DC subtype and methods to generate this subtype in sufficiently high purity; second, the optimal injection route for migration to the peripheral lymphoid organs; and third, the most efficient technique for antigen delivery. The study determined two critical factors in the analyzed clinical trials. In only ten out of 29 studies the DC purity was determined and only in four of those, the DC purity was higher than 80 %. Moreover, DCs fail to express CD62L, a molecule that is essential for homing into the lymph nodes [12].

Although it was initially described that B cells induce tolerance rather than an effector immune response [13, 14], this effect was shown to be limited to resting or lipopolysaccharide (LPS)-activated B cells [15, 16] and seems to depend strongly on the strength of B-cell activation [17]. In contrast, upon stimulation with the CD40 ligand (CD40L) human and murine primary B cells develop into potent antigen-presenting cells (APCs) [18]. CD40B-activated B cells (CD40B cells) can be expanded from small amounts of peripheral blood or splenocytes of healthy donors and cancer patients [19, 20] to an almost unlimited extend [21]. They upregulate costimulatory and MHC molecules and induce naïve and memory CD4^+^ and CD8^+^ T-cell responses in an MHC-restricted manner [19, 20, 22–25]. In contrast to DCs, CD40B cells express all relevant homing molecules, including CD62L, and were shown to migrate to the secondary lymphoid organs *in vivo* in healthy and tumor-bearing mice [26, 27]. *In vivo*, CD40B cells have been demonstrated to induce anti-tumor immunity in a murine breast cancer model [28] and a specific T-cell response in spontaneous cancer in dogs [29, 30].

Recently, the major obstacle for the use of CD40B cells in a clinical trial in cancer patients has been overcome by the development of a soluble human CD40 ligand that can be produced in a GMP-conform manner and induces both strong activation and expansion of human B cells [31].

In the present study, we identified the optimal vaccination parameters for the induction of a specific T-cell response by use of CD40B cells and showed that administration of very high doses of CD40B cells does not induce toxicity *in vivo*. Treatment of mice with peptide-loaded CD40B cells led to anti-tumor immunity resulting in reduced growth of two different tumor entities.

## RESULTS

### CD40L stimulation of murine splenocytes results in the acquisition of an APC phenotype

**Figure 1:**
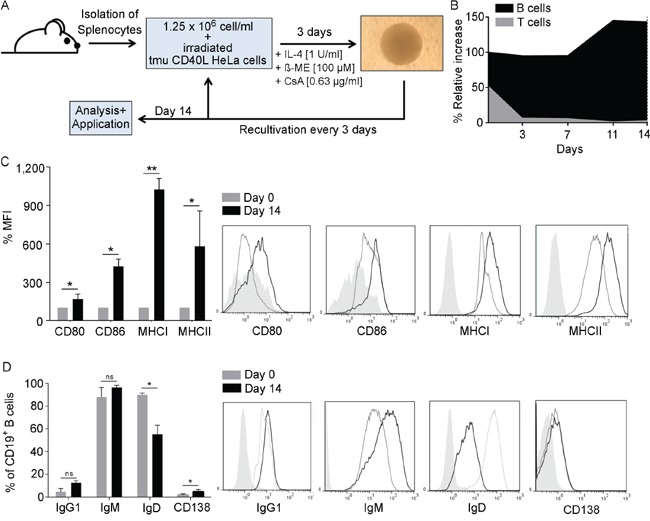
B cells develop an APC-phenotype upon CD40L activation **A**. In the optimal CD40-culture system splenocytes are co-cultured together with CD40L-expressing HeLa cells in the presence of IL-4, ß-mercaptoethanol (ß-ME) and Cyclosporin-A (CsA). Cells are recultivated every 3 days and analyzed or used for experiments after 14 days. **B**. At every recultivation day the relative increase was assessed by normalizing the relative growth to the percentage of CD3^+^ and CD19^+^ cells in the culture as determined by flow cytometry. (n=5). CD40B cells were phenotypically characterized on day 14 by measuring **C**. the mean fluorescence intensity (MFI) of the activations markers CD80 and CD86 and the MHC molecules I and II **D**. or the expression of different immunoglobulin subtypes and CD138. MFI values on day 14 were normalized to values on day 0. Significant differences calculated by Student's t-test are indicated by an asterisk: * p ≤ 0.05, ** p ≤ 0.01, ns = not significant. Histograms show representative analyses compared to an unstained control (filled line). Results of five independent experiments are shown.

Optimized culture conditions for activation of murine splenocytes via CD40L stimulation as described in Figure 1A resulted in the selective proliferation and expansion of CD19^+^ B cells. Over the course of the 14-day culture period B-cell purity steadily increased from ~ 45 % to ~ 98 % (Figure 1B). Treatment with cyclosporine A efficiently inhibited growth of CD3^+^ T cells. On day 14, CD40B cells were analyzed for the expression levels of several surface markers defining their antigen presenting phenotype. CD40B cells highly upregulated the activation markers CD80, CD86, MHC I and MHC II from day 0 to day 14 (Figure 1C) and significantly downregulated the expression of IgD (Figure 1D). The percentage of CD138^+^ plasma cells increased, while the percentage of IgG^+^ or IgM^+^ B cells did not change significantly (Figure 1D).

### CD40B cells induce proliferation and activation of CD4^+^ and CD8^+^ T cells

To study the antigen-presenting function of murine CD40B cells, their ability to stimulate a T-cell response *in vitro* was investigated. Peptide-pulsed APCs from C57BL/6 (B6) mice were co-cultured together with CD4^+^ or CD8^+^ T cells from BALB/C mice. CD40B cells were activated for 7 or 14 days in the CD40L culture. Bone-marrow derived DCs served as alternative source of APCs and positive control in mixed-lymphocyte reactions (MLRs). Maturation of DCs with LPS or CD40L was tested to cover the heterogeneity of DC subsets [32, 33]. Mature DCs highly upregulated the activation markers CD80, CD83, CD86 and IAb (data not shown).

**Figure 2:**
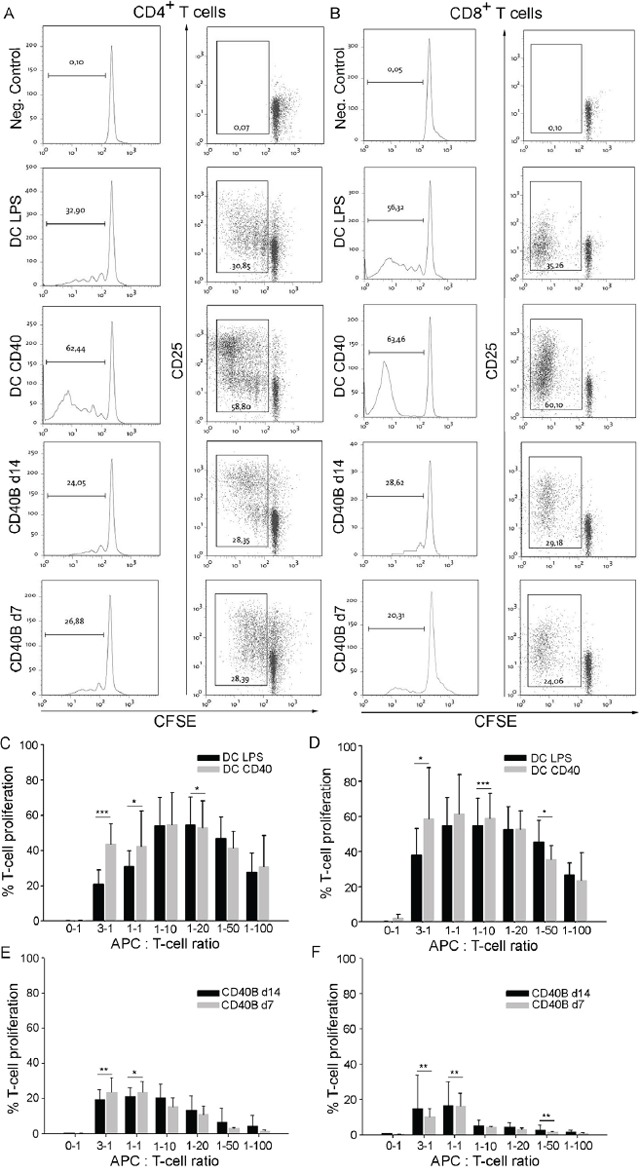
CD40B cells induce T-cell proliferation and activation in allogenic MLRs **A-B**. For negative controls (Neg. Control) T cells were incubated without stimulating APCs. Dendritic cells were stimulated with LPS (DC LPS) or the CD40L (DC CD40). CD40B cells were used on day 7 (CD40B d7) or day 14 (CD40B d14) of activation. Left column= Typical sequential halving of CFSE fluorescence intensity with each generation was detected by flow cytometry. Right column= CFSE intensity, which was accompanied by upregulation of CD25 expression was detected by flow cytometry. One representative experiment out of 8 is shown. **A**. CD3^+^ CD4^+^ T cells from BALB/C mice were cocultured with the indicated APCs. **B**. CD3^+^ CD8^+^ T cells from BALB/C mice were cocultured with the indicated APCs. **C**. CD3^+^ CD4^+^ T-cell proliferation at various APC-to-T cell ratios in allogenic MLRs with either DC LPS or DC CD40 as APCs. **D**. CD3^+^ CD8^+^ T-cell proliferation at various APC-to-T cell ratios in allogenic MLRs with either DC LPS or DC CD40 as APCs. **E**. CD3^+^ CD4^+^ T-cell proliferation at various APC-to-T cell ratios in allogenic MLRs with either CD40B d7 or CD40B d14 as APCs. **F**. CD3^+^ CD8^+^ T-cell proliferation at various APC-to-T cell ratios in allogenic MLRs with either CD40B d7 or CD40B d14 as APCs. Mean values ± SD of eight independent experiments are shown. Significant differences calculated with Student's t-test are indicated by an asterisk: * p ≤ 0.05, ** p ≤ 0.01, *** p ≤ 0.001.

T-cell activation and proliferation was determined by flow cytometry. In an APC-to-T cell ratio of 1:1, both LPS- and CD40L-matured DCs induced high proliferation and activation of CD4^+^ (Figure 2A) and CD8^+^ T cells (Figure 2B). In all tested APC-to-T cell ratios, LPS-matured DCs were less potent in the induction of a CD4^+^ or CD8^+^ T-cell response than CD40L-matured DCs (Figure 2C and 2D, respectively). As expected from their expression of activation markers, CD40B cells were highly potent in the initiation of an alloreactive CD4^+^ or CD8^+^ T-cell response by inducing both proliferation and activation of the T cells (Figure 2A and 2B). However, even in high B-to-T cell ratios they induced less proliferation than LPS- or CD40L-matured DCs. While DCs induced four to five rounds of division in CD4^+^ and CD8^+^ T cells, CD40B cells only induced two to three rounds of division. Interestingly, at high APC-to-T cell ratios, CD40B cells that were activated for 7 days only (CD40B d7) induced significantly more proliferation of CD4^+^ T cells than CD40B cells that were activated for 14 days (CD40B d14) (Figure 2E). In contrast, the proliferation of CD8^+^ T cells was higher when cultured together with CD40B d14 (Figure 2F). With a decreasing CD40B-to-T cell ratio the proliferation of T cells decreased. This effect was also observed in DC cultures, but less pronounced.

### Adoptive transfer of CD40B cells does not lead to toxicity *in vivo*

**Figure 3:**
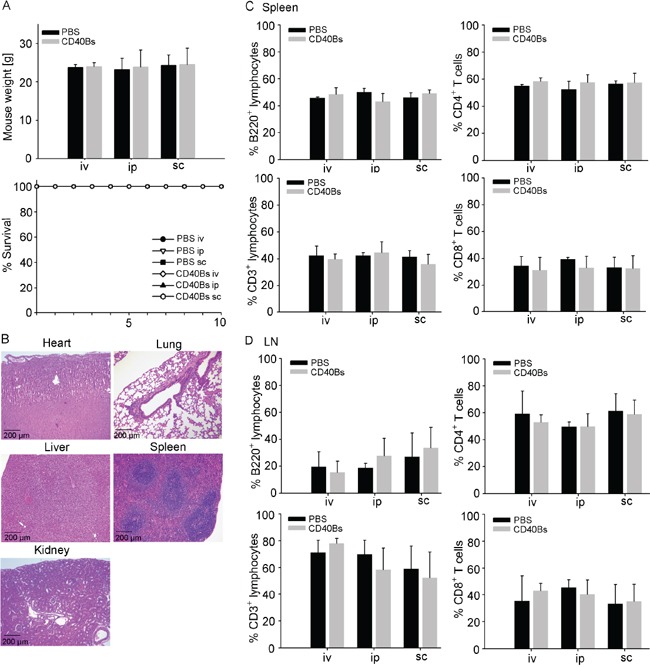
Immunization with CD40B cells does not lead to *in vivo* toxicity B6 mice were challenged with either CD40B cells or PBS alone as negative control in three different routes; i.e. intravenous (iv), intraperitoneal (ip) or subcutaneous (sc). Five weeks after CD40B cell injection they were analyzed for indications of toxicity. **A**. The weight of the mice and the survival over a period of ten days were documented. Mean values ± SD of four independent experiment with five mice per group are shown. **B**. H&E stained sections of heart, lung, liver, spleen and kidney were analyzed. Representative pictures out of four independent experiments are shown. **C**. Spleens of mice were analyzed by flow cytometry for B220^+^ B cells or CD3^+^ T cells and the T cell subpopulations CD4^+^ and CD8^+^ cells. Mean values ± SD of four independent experiment with five mice per group are shown. **D**. Mesenteric and inguinal lymph nodes (LN) of mice were analyzed by flow cytometry for B220^+^ B cells or CD3^+^ T cells and the T-cell subpopulations CD4^+^ and CD8^+^ cells. Mean values ± SD of four independent experiment with five mice per group are shown.

Toxic side effects are a critical point when considering adoptive transfer of immune cells for the clinical application. To rule out the possibility of toxic side effects from adoptive transfer of CD40B cells, we challenged B6 mice with pure CD40B cells in three different injection routes, i.e. intraperitoneal (ip.), intravenous (iv.) and subcutaneous (sc.). In lack of a murine soluble CD40L that induces both strong activation and expansion of murine B cells, CD40B cells were generated with CD40L-expressing feeder cells as described in Figure 1A. However, this activation system is not clinically applicable in studies with humans. To address long-term toxicity, twice per week over a period of five weeks we injected high doses of CD40B cells, i.e. 1 × 10^6^ cells/injection equal to 40 × 10^6^ cells/kg. Injection of PBS buffer served as negative control. After five weeks of further observation, mice were analyzed for changes in behavior, weight or survival. In all three injections routes we could not detect any differences compared to controls (Figure 3A). Furthermore, we performed histo-pathological analyses of clinical relevant organs (Figure 3B) and screened secondary lymphoid organs for rejection reactions (Figure 3C and 3D). A detailed examination of heart, lung liver, spleen and kidney sections revealed no abnormal lymphocytic infiltration, structural tissue injury or other indications of inflammation (Figure 3B). Spleens (Figure 3C) as well as mesenteric and inguinal lymph nodes (Figure 3D) were analyzed for changes in the lymphocyte subsets by flow cytometry. No significant differences were observed in the percentage of B220^+^ B cells or CD3^+^ T cells and the T-cell subpopulations CD4^+^ T cells and CD8^+^ T cells between controls and CD40B cell-treated mice. Acute toxicity was assessed by treating mice once with a very high dose of CD40B cells, i.e. 1 × 10^7^ cells/injection equal to 40 × 10^7^ cells/kg. No abnormal changes in organ sections or flow cytometry analyses of lymphoid organs were observed (data not shown). In comparison, in clinical trials with DC-based vaccines in patients with prostate and renal cell cancer average doses of 0.06 – 0.13 × 10^6^ cell/kg were administered [34–42] with the lowest dose of 0.0075 × 10^6^ cell/kg [43] and the highest dose of 1.5 × 10^6^ cell/kg [44]. In a study with dogs with spontaneous cancer 1-5 × 10^6^ CD40B cells were injected per dog, which equals 0.03-0.15 × 10^6^ cells/kg [30]. Our data therefore demonstrate the safety of CD40B-cell administration for *in vivo* adoptive transfer.

### CD40B cells induce an antigen-specific CD8^+^ T-cell response *in vivo*

Since CD40B cells showed the capacity to induce T-cell responses *in vitro*, we tested their antigen-presenting function *in vivo*. In addition, we evaluated different vaccination parameters, including the injection route, the cell dose and injection intervals. Induction of specific CD8^+^ T-cell responses was assessed using two different functional assays. First, the direct cytolytic activity was determined by measuring the specific lysis of peptide-pulsed target cells in spleens of APC-vaccinated B6 mice. Second, the percentage of IFN-γ producing CD8^+^ T cells was analyzed by flow cytometry in spleens of APC-vaccinated mice. To increase signal strength OT-I mice were used in this experiment instead of B6 mice.

**Figure 4:**
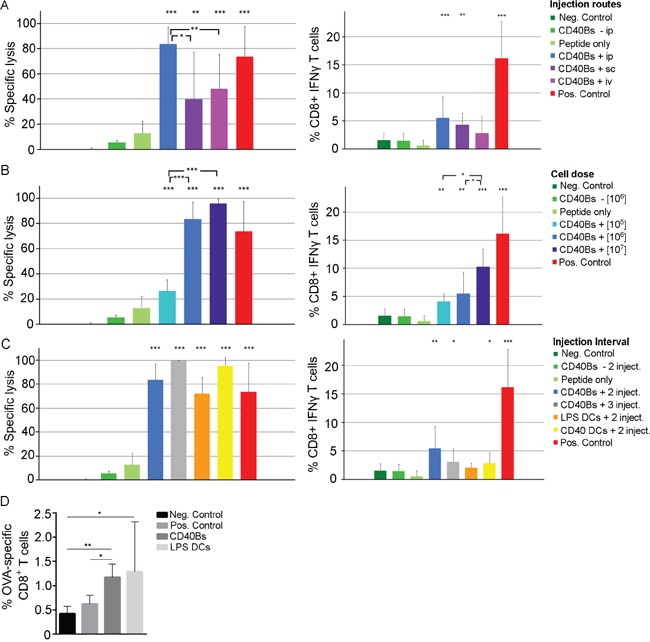
Immunization with CD40B cells induces specific T-cell responses *in vivo* **A-C**. Left column = Specific lysis was determined by injecting 1 × 10^7^ target cells labeled with different amount of CFSE ip. into vaccinated B6 mice 4 days after the last immunization. The ratio of OVA-pulsed target cells versus unpulsed target cells was determined by flow cytometry one day later and the specific lysis was calculated. Right column = Production of IFN-γ was determined by flow cytometry after restimulating purified lymphocytes of vaccinated OT-I mice with peptide. Mean values of frequencies of restimulated cells were normalized to corresponding values of unstimulted cells. Mice were immunized with OVA-peptide pulsed CD40B cells (CD40B +) or unpulsed CD40B cells (CD40 -). Negative controls (Neg. Control) were immunized with PBS+IFA alone, positive controls (Pos. Control) were immunized with Peptide + CpG + IFA. Bar charts show mean values ± SD of four independent experiments with three mice per group. Significant differences calculated with Student's t-test are indicated by an asterisk: * p ≤ 0.05, ** p ≤ 0.01, *** p ≤ 0.001. **A**. Three different injection routes were tested for vaccination with peptide-pulsed CD40B cells; i.e. intravenous (iv), intraperitoneal (ip) or subcutaneous (sc). **B**. Different cell numbers were tested for vaccination with peptide-pulsed CD40B cells; i.e. 1 × 10^5^, 1 × 10^6^ and 1 × 10^7^. **C**. Different vaccination intervals were tested for vaccination with peptide-pulsed CD40B cells; i.e. injection on day 0 and 7 (2 inject.) or on day 0, 7 and 14 (3 inject.). Peptide-pulsed DCs stimulated with LPS or CD40L served as additional control. **D**. CD3^+^ CD8^+^ T-cells from spleens of vaccinated B6 mice were stained for the presence of OVA peptide-specific T-cells. Bar charts show mean values ± SD of four independent experiments with three mice per group. Significant differences calculated with Student's t-test are indicated by an asterisk: * p ≤ 0.05, ** p ≤ 0.01.

Significant differences in the induction of specific lysis were observed when testing different injection routes (Figure 4A) by vaccinating mice once with 1 × 10^6^ CD40B cells. The cytolytic activity of CD8^+^ T cells was significantly higher in mice that were vaccinated ip. than in mice that were vaccinated sc. or iv. No significant differences were observed in the percentage of IFN-γ CD8^+^ T cells between the different injection routes. However, the percentage of IFN-γ producing CD8^+^ T cells was higher in mice that were vaccinated with peptide-pulsed CD40B cells than in mice that were treated with unpulsed CD40B cells or peptide alone. Henceforward, in all subsequent experiments mice were vaccinated ip.

We next determined the optimal cell dose for vaccination. A dose-dependent effect of vaccinations with CD40B cells was identified. An increase of the cell dose from 1 × 10^5^ cells/injection to 1x 10^6^ cells/injection led to a significant increase in the induction of specific lysis and the production of IFN-γ in CD8^+^ T cells (Figure 4B). Even though a further increase of cell dose 1x 10^6^ cells/injection to 1x 10^7^ cells/injection did not result in significant differences in the specific lysis it led to an increase in the production of IFN-γ CD8^+^ T cells.

To determine the effect of varying vaccination intervals, mice received either two or three injections of 1 × 10^6^ cells ip. in an interval of seven days. LPS- or CD40L-stimulated DCs were included into the vaccination experiments to compare their antigen-presenting function to that of CD40B cells. Following a second booster vaccination the percentage of specific lysis increased from 83.7 % ± 13.4 with one booster vaccination to 99.8 % ± 0.3 with two booster vaccinations (Figure 4C). This effect was not reflected in an increase in IFN-γ production. The levels of specific lysis and IFN-γ CD8^+^ T cells were comparable in mice that were vaccinated with LPS- or CD40L-matured DCs.

The induced T-cell response after vaccination with protein-pulsed APCs was characterized by staining CD3^+^ T cells for the presence of ovalbumin (OVA)-peptide specific CD8^+^ T cells (Figure 4D). Mice that were vaccinated with CD40B cells showed significantly higher percentages of OVA-specific CD8^+^ T cells than negative and positive controls, but lower percentages than mice that were treated with mature DCs. No increase in CD25 expression was observed (data not shown).

### Treatment with CD40B cells induces anti-tumor immunity *in vivo*

**Figure 5:**
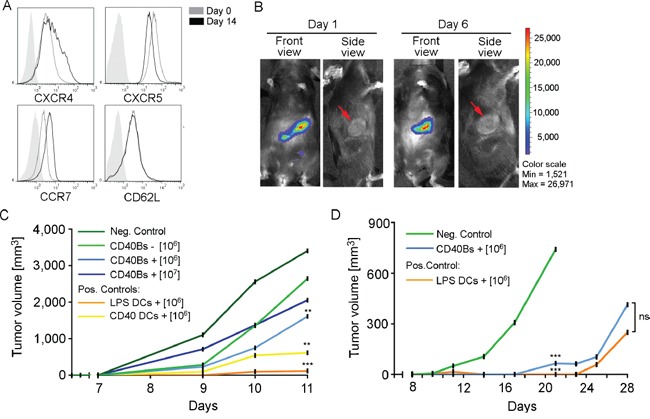
Vaccination with CD40B cells induces anti-tumor immunity *in vivo* **A**. Expression of the homing molecules CXCR4, CXCR5, CCR7 and CD62L in CD40B cells from luciferase^+^ mice on day 14 was compared to the expression before stimulation. Histograms show representative analyses of four independent experiments compared to an unstained control (filled line). **B**. CD40B cells from luciferase^+^ mice were injected intraperitoneally into E.G7 tumor-bearing B6 mice. Homing behavior was measured in the IVIS system on day 1 or day 6 after injection. Representative pictures of five independent experiments with three mice per group are shown. **C**. B6 mice were treated with 1 × 10^6^ or 1 × 10^7^ TRP2-peptide pulsed CD40B cells (CD40Bs +) or unpulsed CD40B cells (CD40Bs -) three times in an interval of seven days before challenging them with B16.F10 melanomas 7 days after the last vaccination. Alternatively, mice were vaccinated with 1 × 10^6^ LPS or CD40L stimulated DCs. Injection of PBS alone served as negative control (Neg. Control). Tumor growth was assessed daily. One representative experiment out of two independent experiments with three mice per group is shown. Significant differences calculated with paired Student's t-test are indicated by an asterisk: ** p ≤ 0.01, *** p ≤ 0.001. **D**. B6 mice were treated with 1 × 10^6^ OVA-protein pulsed CD40B cells (CD40Bs +) three times in an interval of seven days before challenging them with E.G7 lymphomas 7 days after the last vaccination. As positive control, mice were vaccinated with 1 × 10^6^ LPS stimulated DCs. Injection of PBS alone served as negative control (Neg. Control). Tumor growth was assessed daily. One representative experiment of four independent experiments with three mice per group is shown. Significant differences calculated with paired Student's t-test are indicated by an asterisk: ns = not significant, *** p ≤ 0.001.

The effect of a preventive treatment with CD40B cells on tumor growth was determined by challenging mice with B16.F10 melanomas or E.G7 lymphomas. To determine the migratory behavior after ip. injection, CD40B cells that were generated from luciferase^+^ mice were injected into E.G7 tumor-bearing B6 mice and their migration was tracked using the IVIS system. CD40B cells showed the expected migratory pattern (Figure 5A) [26]. One and five days after ip. injection luciferase^+^ CD40B cells were detected in the spleen and the tumor-draining lymph nodes, but not the tumor itself (Figure 5B). To examine the effect of peptide/protein-loaded CD40B cells in a preventive vaccination approach for tumor targeting, 10^6^ peptide/protein-pulsed APCs were injected into B6 mice ip. three times in an interval of seven days. On day 21, immunized mice were challenged with 1 × 10^6^ B16.F10 melanoma cells (Figure 5C) or 0.4 × 10^6^ E.G7 lymphoma cells (Figure 5D). Tumor growth of B16.F10 melanomas in mice that were treated with 1 × 10^6^ CD40B cells/injection was significantly reduced compared to negative controls on day 11, but only when cells were pulsed with B16 melanoma-specific TRP2-peptide (Figure 5C). An increase of the cell dose 1 × 10^7^ CD40B cells/injection did not result in a significant difference in tumor growth compared to the lower cell dose. Tumors in mice that were treated with LPS- or CD40L-stimulated DCs grew slower than in CD40B-cell treated mice. Two of three mice that were treated with LPS-stimulated DCs stayed tumor free until day 13 after tumor challenge (data not shown).

For the E.G7 lymphoma model, mice were vaccinated with OVA-protein pulsed APCs. Only the optimal vaccination conditions for CD40B cells and dendritic cells, i.e. 10^6^ CD40B cells and LPS-activated DCs, were chosen for this experiment. E.G7 tumors grew slower than B16.F10 melanomas in general and negative controls survived until day 20 (Figure 5D). At this time point, APC-treated mice showed a significantly reduced tumor growth compared to negative controls. Mice that were treated with CD40B cells or DCs survived until day 28 and there was no significant difference in tumor volume.

## DISCUSSION

Our results presented in this study support the potential of CD40B cells for cancer immunotherapy and underline the importance of the optimal vaccination strategy for cellular immunotherapy.

So far contradicting results were described about the antigen-presenting capacity of CD40B cells *in vivo*. The discrepancy between our own results and the studies that reported weak T-cell responses by CD40B-cell vaccination can be explained by the different setup of the studies. Lee et al. [45] and Guo et al. [46] used monoclonal anti-CD40 antibodies to activate their B cells, which did not result in the same elevated expression levels of costimulatory and MHC molecules that we observed in our study by using CD40L-expressing feeder cells for the activation of B cells. This effect is probably due to higher cross-linking capacity of membrane-bound CD40L [31, 47, 48]. Our results are supported by a study in 4T1 breast cancer tumors [28] that demonstrated an induction of a specific T-cell response by CD40B-cell vaccination *in vivo* and a reduced appearance of metastases. It was also suggested that peptide-pulsing of CD40B cells is not sufficient for antigen presentation and the induction of a T-cell response [28, 46]. However, failure of peptide-pulsed B cells to induce a specific T-cell response probably also resulted from insufficient activation of B cells and the failure to upregulation costimulatory molecules. Our results obtained here clearly demonstrate that peptide-pulsing of highly activated CD40B cells is indeed sufficient to induce an effective immune response. We thereby offer a simpler method for the development of efficient APCs, since other methods for stable presentation of antigen by B cells is critical in a human application. Furthermore, we showed that the *in vivo* induction of the CD8^+^ T-cell response depends on the injection route and the dose of injected cells. It seems that even at low numbers under optimal vaccination conditions and after strong CD40L-activation, CD40B cells overcome inhibitory mechanisms by the tumor. This idea is also supported by a study on tumor-mediated immune suppression of CD40B cells. Phenotype, migratory potential and antigen-presenting function of human CD40B cells were shown to be resistant to interleukin-10, TGF-β and VEGF [48].

Under optimal vaccination conditions, CD40B cells showed similar or less effect on tumor growth than mature DCs. Nevertheless, DCs should not simply be identified as superior APCs for cancer immunotherapy. Some aspects make the comparison of two such very fundamentally differing cell types difficult. DCs possess a 4.5 times higher surface area than CD40B cells (data not shown) and therefore express more costimulatory and MHC molecules on their surface. Since both DCs and CD40B cells were shown to make contact with more than one T cell at a time [26], a higher surface area might lead to an advantage in antigen presentation when the same number of APCs are used. To truly compare the two cell types, cell numbers adjusted for surface size should be used. Moreover, maturation of DCs by LPS might not be the optimal stimulus, since LPS is a known to induce a septic shock in humans and mice [49]. In addition, CD40B cells and DCs were administered in different injections routes to apply both cell types under their most optimal conditions.

Similar to DCs, B cells are able to take up antigen by fluid phase pinocytosis. However, their strength lies in uptake of a specific antigen via the B cell receptor (BCR). The BCR has a high affinity for a given antigen and allows B cells to concentrate very small quantities of their specific antigen resulting in important changes in the antigen processing machinery [50]. In contrast, presentation after uptake by fluid phase pinocytosis in B cells requires about 5000 times higher concentrations [51]. Therefore, it might be possible to enhance CD40B-cell vaccination by use of tumorantigen-specific CD40B cells.

In a study with dogs with spontaneous cancer, one dog developed signs of acute systemic reaction. However, no long-term complications were monitored [30]. In our study, application of CD40B cells even in very high cell doses that were 3000 to 6000 times higher than cell doses used in clinical DC-based studies or pre-clinical CD40B-cell studies proved to be safe without any toxic side effects. This is highly relevant since the induction of clinical toxicity is still a major problem in current cellular immunotherapies [52]. The choice of antigen for loading of CD40B cells is of crucial importance and should be limited to tumor-specific or tumor-associated antigens only. Here, we did not load CD40B cells with antigen for toxicity assessment, but mice in tumor control experiments showed no signs of autoimmune reaction. Additionally, the existence of the clinically approved anti-CD20 antibody Rituximab offers the option to deplete B cells if the induced immune reaction should lead to autoimmunity [53].

Taken together, CD40B cells were shown to induce anti-tumor immunity even in the low immunogenic model B16.F10 that expresses minimal PD-L1 [54]. Active tumor antigen-specific vaccination with CD40B cells therefore might be a promising combination partner for cancer immunotherapy in conjunction with checkpoint inhibitors. With regard to the earlier mentioned challenges in cellular immunotherapy, CD40B cells with an activated phenotype can be easily generated in high purity, a clinical applicable injection route results in strong T-cell responses and B cells take up whole protein antigen by simple protein pulsing. CD40B cells therefore might induce weaker immune responses *in vivo* than mature DCs, but their use in the clinic promises to be more feasible.

## MATERIALS AND METHODS

### Ethics statement

Investigation has been conducted in accordance with the ethical standards and according to national and international guidelines and has been approved by the authors’ institutional review board.

### Blood samples and mice

C57BL/6 mice (designated as B6) and BALB/C mice were obtained from JANVIER Labs. Luciferase+ C57BL/6 mice were kindly provided by Prof. Dr. Robert Zeiser (Laboratory for Allo-Immunregulation, Department for Internal Medicine I, University Hospital Freiburg, Germany). OT-I mice expressing OVA-specific CD8^+^ T cells were obtained from the Jackson Laboratory. Mice were housed under specific pathogen-free conditions and all animal experiments were approved by the Cologne regional animal care committee.

### Isolation of lymphocytes

Murine splenocytes were isolated using Pancoll density-gradient centrifugation (Pan-Biotech). B cells were enriched using positive immunomagnetic selection with murine CD19 microbeads (Miltenyi Biotec) according to manufacturer's protocol. Murine T cells were purified by negative selection using EasySep Mouse T Cell Enrichment Kit (Stem Cell Technologies) according to manufacturer's protocol.

### Generation of murine CD40B cells

Murine B cells were activated and expanded using the established system of adherent murine epithelial cell lines expressing murine CD40L. tmuCD40L HeLa feeder cells were kindly provided by Professor Clemens Wendtner (Klinikum Schwabing, Munich, Germany). Cell culture was performed as described previously [55]. Briefly, CD19^+^ B cells or splenocytes were seeded at a density of 1 × 10^6^ cell/ml on lethally irradiated feeder cells. Cell passaging of feeder cells was done twice per week. B cells were cultured in DMEM medium (Life Technologies) supplemented with 580 μg/ml Glutamine, 10% Fetal-bovine serum, 1 % HEPES, 1 % MEM and 15 μg/ml gentamicin. 50U/mL of recombinant murine IL-4 (Immunotools) were freshly added.

### Generation of mature dendritic cells

Murine CD34^+^ progenitor cells were purified from bone marrow of hind limbs of B6 mice by positive selection with EasySep Biotin Selection Kit (Stem Cell Technologies) using an anti-murine CD34-biotinylated antibody (Biolegend). The cells were cultivated at a concentration of 0.25 × 10^6^ cell/ml in VLE-RPMI medium supplemented with 5 % FBS, 50 μM ß-mercaptoethanol and 500 U/ml of murine granulocyte macrophage colony-stimulating factor and 1 U/ml murine IL-4. On days 3, and 5 of the cultivation period, the medium was replaced. For maturation, the medium was supplemented with either 10 ng/ml LPS (Sigma Aldrich) or 1 μg/ml anti-mouse CD40L antibody (clone HM40-3, Acris Antibodies).

### Flow cytometry

Cell phenotypes were evaluated using the following fluorophore-conjugated antibodies for the indicated cell types: B cells with anti-mouse B220, CD19 (Life Technologies), CD80, CD86, CD138, IA^b^, IgD, IgG1, IgM and H2K^b^ (Biolegend); T cells with CD3e, CD4, CD8, CD25, CD62L (Biolegend) and SIINFEKL-H2K^b^ Tetramer (Glycotope Biotechnology); DCs with CD11b, CD11c, CD80, CD83, CD86 (Biolegend). 0.5×10^6^ cells were washed twice with PBS and incubated with antibodies for 15 min at 4 °C. The data were collected on a Gallios flow cytometer (Beckman Coulter) and analyzed using FlowJo (Tree Star) or Kaluza (Beckman Coulter) software.

### Intracellular staining for INF-γ

Splenocytes from vaccinated mice were purified by Pancoll and stimulated with 10 μM OVA-peptide for 4 hours at 37 °C. Stimulation with Paramethoxyamphetamin and Ionomycin served as positive control. Incubation was stopped by adding 0.66 μl Golgi-Stop/10^6^ cells (BD Biosciences). Cells were stained for surface markers before they were fixed with 250 μl Cytofix/Cytoperm solution (BD Biosciences). After 20 min incubation, they were permeabilized by incubation in 500 ml Perm/Wash solution. Ten min later, cells were stained for intracellular IFN-γ. Mean values of frequencies of IFN-γ^+^ CD8^+^ T cells that were restimulated with peptide were normalized to corresponding unstimulated samples.

### Mixed-lymphocytes reactions

APCs were irradiated once with 26 Gy and then mixed at different ratios with CD3^+^ T cells from spleens of BALB/C mice. T cells were stained with the 10 μM CFSE. Co-cultures were incubated for 5 days.

### *In vivo* immunization of naïve mice

For positive controls B6 mice were immunized with OVA-Protein/IFA solution in addition of 7 μM immunomodulatory CpG-ODN 2395 (oligodeoxynucleotide with non-methylated cytosine-guanine motifs). For immunization in the E.G7 tumor model, APCs were exogenously loaded with 10 μM OVA-protein for 1 hour at 37 °C. For immunization in the B16.F10 melanoma model, APCs were loaded with TRP-2 peptide (aa180-188). For *in vivo* cytotoxicity assays and IFN-γ assays, APCs were loaded with OVA-peptide (aa257-264).

### *In vivo* cytotoxicity assay

For *in vivo* cytotoxicity assays, OVA-peptide (aa257-264) pulsed CFSE-labeled target cells were injected i.p. into immunized mice on day 21. Twenty-four hours later, spleens were removed and analyzed for killing of peptide-pulsed target cells. The ratio of unpulsed versus pulsed (RatioUP) target cells was determined by dividing the percentage of CFSE low cells by the percentage of CFSE high cells. Specific lysis was calculated by the following formula: % Specific Lysis = (1-(RatioUP Negative Control/RatioUP Immunized))*100.

### *In vivo* migration

Murine CD40B cells from luciferase+ mice were injected ip. into B6 mice. Detection of luciferase^+^ B cells was performed by injecting 7.5 mg D-Luciferin in 250 ml 1x PBS i.p. The luciferin was allowed to distribute in the mouse for 5 min before mice were narcotized with 1.5-4 % Insofluran. Mice were shaved prior to imaging in order to minimize interference by the fur. Imaging was performed in the Xenogen IVIS 200 (Perkin Elmer). Mice were constantly kept under narcosis with 1.5-4 % Insofluran at 37 °C. Bioluminescence pictures were analyzed with the Living Image Software (Perkin Elmer).

### Assessment of secondary immunological effects

B6 mice were immunized with 1 × 10^7^ CD40B cells i.v., i.p. or s.c. twice per week. Mice were observed for changes in behavior and clinical symptoms, i.e. reduced food and water intake, reduced activity, difficulties of breathing and moving and increased body temperature. After five weeks mice were sacrificed, weighted and organs analyzed by H&E staining.

### Tumor challenge

Tumor cells E.G7 and B16.F10 were kept at a low concentration of 1 × 10^6^ cells/ml prior to injection. For tumor formation, cells were harvested and resuspended at a concentration of 0.4 × 10^7^ cells/ml or 1 × 10^7^ cells/ml in 1x PBS, respectively. 100 μl were injected s.c. into the right flank of immunized or naïve B6 mice. Tumor size was determined daily from day 7 after inoculation by measuring tumor diameter in two dimensions using a vernier caliper. The tumor volume was calculated using the following formula: Tumor volume = 0.5 x (length x width^2^) mm. Tumors were allowed to grow for 40 days or until one diameter reached a size of 15 mm.

### Statistical analysis

Significant differences were calculated by Student's t-test for paired data, ordinary one-way ANOVA or ordinary two-way ANOVA were appropriate using GraphPad Prism Software. P-values of less than 0.05 were considered statistically significant and marked with asterisks: * p ≤ 0.05, ** p ≤ 0.01, *** p ≤ 0.001, **** p ≤ 0.0001. Mean values and standard deviations (SD) were calculated from at least 3 independent experiments.
